# Magnesium intake and mortality due to liver diseases: Results from the Third National Health and Nutrition Examination Survey Cohort

**DOI:** 10.1038/s41598-017-18076-5

**Published:** 2017-12-20

**Authors:** Lijun Wu, Xiangzhu Zhu, Lei Fan, Edmond K. Kabagambe, Yiqing Song, Menghua Tao, Xiaosong Zhong, Lifang Hou, Martha J. Shrubsole, Jie Liu, Qi Dai

**Affiliations:** 1 Department of Digestive Diseases of Huashan Hosptial, Fudan University, Shanghai, 200040 China; 2 Division of Epidemiology, Department of Medicine, Vanderbilt University Medical Center, Nashville, TN 37203 USA; 3 Vanderbilt Ingram Cancer Center, Vanderbilt University School of Medicine, Nashville, TN 37232 USA; 4 Department of Oncology, Longhua Hospital, Shanghai University of Traditional Chinese Medicine, Shanghai, 200437 China; 5 Department of Epidemiology, Richard M. Fairbanks School of Public Health, Indiana University, Indianapolis, IN 46202 USA; 6 Department of Biostatistics and Epidemiology, University of North Texas Health Science Center, Fort Worth, TX 76107 United States; 7 Cancer Center, Beijing Shiji Tan Hospital, Capital Medical University, Bejing, 100038 China; 8 Department of Preventive Medicine, Feinberg School of Medicine, Northwestern University, 680 N. Lake Shore Drive, Suite 1400, Chicago, IL 60611 USA

**Keywords:** Magnesium, Liver diseases

## Abstract

People with fatty liver disease are at high risk of magnesium deficiency. Meanwhile, low magnesium status is linked to both chronic inflammation and insulin resistance. However, no study has investigated the association between intake of magnesium and risk of mortality due to liver diseases. We evaluated the association between total magnesium intake and mortality due to liver diseases in the Third National Health and Nutrition Examination Study (NHANES III) cohort, which included 13,504 participants who completed liver ultrasound examination for hepatic steatosis. Overall magnesium intake was associated with a reduced risk of mortality due to liver disease at borderline significance (P = 0.05). In fully-adjusted analyses, every 100 mg increase in intake of magnesium was associated with a 49% reduction in the risk for mortality due to liver diseases. Although interactions between magnesium intake and alcohol use and hepatic steatosis at baseline were not significant (P > 0.05), inverse associations between magnesium intake and liver disease mortality were stronger among alcohol drinkers and those with hepatic steatosis. Our findings suggest higher intakes of magnesium may be associated with a reduced risk of mortality due to liver disease particularly among alcohol drinkers and those with hepatic steatosis. Further studies are warranted to confirm the findings.

## Introduction

All-cause cirrhosis and liver cancer are two of the four top leading causes of death from gastrointestinal and liver disease in United States^1^ and globally, the mortality rate due to liver cirrhosis has increased over the past 35 years^2^. Nonalcoholic fatty liver disease (NAFLD), alcoholic liver disease, and hepatitis B and C infection are the major causes for liver cirrhosis and cancer^3^. NAFLD is the most common liver disease in the world^4^, which includes a spectrum of liver injury ranging from steatosis to severe steatohepatitis that can progress to fibrosis, cirrhosis, liver failure, or even liver cancer^5^. Unlike alcoholic liver disease and viral infections, the etiology of NAFLD remains poorly understood and effective interventions are scant. However, NAFLD is considered a feature of metabolic syndrome^6^. Inflammation^7^ and insulin resistance^8,9^ play critical roles in the progression of NAFLD. Likewise, inflammation and insulin resistance also may occur and aggravate alcoholic liver disease^10,11^ and hepatitis C and B infection^12,13^.

One previous study found that serum magnesium levels were significantly lower in patients with either alcoholic or non-alcoholic liver steatosis^14^. People who chronically drink alcohol are at high risk of magnesium deficiency^15–17^. It has been found that prolonged exposure to alcohol leads to a substantially altered homeostasis of magnesium in the liver^18^. In addition to alchol drinkers, as much as 50% of type 2 diabetic patients had low serum magnesium levels^19^. Magnesium deficiency causes inflammation^20,21^, while randomized trials conducted among those at high risk of inflammation (i.e. obese women^22^, heart failure patients^23^ or those with high-sensivity C-reactive protein >3 mg/l^24^) found magnesium treatments reduced hs-CRP concentrations. Moreover, a meta-analysis of randomized trials indicates magnesium supplementation improves insulin resistance in type 2 diabetes patients^25^.

Thus, we hypothesized that higher intakes of magnesium will be associated with a reduced risk of mortality due to liver disease regardless of alcohol use or hepatic steatosis at baseline. To test this novel hypothesis, we analyzed the data from the Third National Health and Nutrition Examination Survey (NHANES III) follow-up cohort.

## Results

The individuls with fatty liver at baseline were more like to be older, physically active, men, non-Hispanic black, obese, former smokers, and former alcohol drinkers, and to have lower educational chievement and annual household income in comparison to those without fatty liver disease (Table 1). There were no significant differences in intakes of of total energy, total magnesium, and total calcium between individuals with and without fatty liver disease.

**Table 1:** Baseline demographic and selected risk factors by the status of hepatic steatosis, the Third National Health and Nutrition Examination Survey (NHANES III)^a^.

	Moderate/Severe HS	Normal/Mild HS	*p*-value^b^
	n = 3081	n = 10423	
**Age at Screening (years)**			
Mean (95% CI)	47.1 (46.1–48.1)	40.9 (40.1–41.7)	<0.0001
Gender, %			<0.0001
Male	1565 (53.7)	4726 (46.8)	
Female	1516 (46.3)	5697 (53.2)	
Race/Ethnicity, %			0.0015
Non-Hispanic White	1075 (74.8)	3849 (75.9)	
Non-Hispanic Black	692 (9.2)	3269 (11.4)	
Other Race	1314 (16.0)	3305 (12.8)	
Education Level, %			<0.0001
Less Than High School	1356 (29.4)	3462 (20.8)	
High School (including GED)	906 (35.8)	3384 (34.6)	
Some College or Above	695 (34.8)	3283 (44.6)	
Poverty income ratio (PIR), %			0.055
≤1	744 (14.3)	2185 (12.1)	
1–3	1312 (43.6)	4376 (41.1)	
>3	734 (42.1)	2984 (46.8)	
Body Mass Index (kg/m^2^)			<0.0001
Mean (95% CI)	30.3 (29.8–30.9)	25.6 (25.4–25.8)	
**Smoke status, %**			
Non smoker	1430 (42.0)	5152 (46.0)	<0.0001
Former smoker	902 (33.5)	2200 (22.8)	
Current Smoker	749 (24.6)	3070 (31.2)	
**Alcohol drinking, %**			
Non drinker	508 (13.1)	1673 (11.9)	0.0003
Former drinker	1128 (35.8)	3479 (30.0)	
Current drinker	1380 (51.1)	5055 (58.1)	
Exercise, %			0.0008
Less active than others	768 (24.8)	2252 (21.3)	
Same as others	1477 (47.0)	4795 (44.7)	
More active than others	767 (28.2)	3227 (34.0)	
**Daily nutrients intake, Mean (95% CI)**			
Energy (kcal)	2465.9 (2175.7–2756.0)	2729.6 (2371.8–3087.3)	0.24
Total calcium intake (mg)	866.5 (823.0–910.0)	873.0 (845.7–900.4)	0.73
Total magnesium intake (mg)	320.7 (309.1–332.3)	323.4 (316.7–330.0)	0.65

^a^Values presented are unweighted frequencies (weighted percentage, %) or weighted mean (95% CI).

^b^Rao-Scott chi-square test for categorical data, and survey regression model for continuous variables.

We observed that high total intake of magnesium was significantly associated with a reduced risk of mortality due to liver disease in models 1 although the association became of borderline significance (p = 0.05) after further adjusting for other potential confounders. There was a 49% reduction in risk of death from liver disease for every 100 mg increase in magnesium intake. Tests for interactions between magnesium intake and potential effect modifiers namely fatty liver status (p = 0.84) and alcohol drinking status (p = 0.86) were not significant. However, the inverse associations between magnesium and death from liver disease were significant among those with fatty liver disease (HR = 0.35, 95% CI = 0.14–0.89) and of borderline significance among alcohol drinkers (HR = 0.46, 95% CI = 0.22–0.99) (Table 2). Similar findings were found in analyses by quantiles (including tertiles and quartiles). We did not find calcium intake was significantly related to the risk of mortality due to liver disease.

**Table 2:** Magnesium intake and hazard ratios (95% CIs) for mortality due to liver disease stratified by status of hepatic steatosis or alcohol drinking at baseline, Third National health and Nutrition Examination Survey 1988–1994^a,b,c^.

Total	Model 1	Model 2	Model 3
	N = 13504	N = 13442	N = 11545
HR per 100 mg/d (95% CI)	0.59 (0.38, 0.93)	0.54 (0.30–1.00)	0.51 (0.26–1.01)
*P*-value	0.02	0.05	0.05
No hepatic steatosis	N = 10423	N = 10371	N = 8979
HR per 100 mg/d (95% CI)	0.54 (0.30, 0.99)	0.56 (0.25, 1.21)	0.57 (0.23–1.39)
*P*-value	0.05	0.14	0.21
Hepatic steatosis	N = 3081	N = 3071	N = 2566
HR per 100 mg/d (95% CI)	0.69 (0.36, 1.31)	0.53 (0.25–1.11)	0.35 (0.14–0.89)
*P*-value	0.25	0.09	0.03
Never alcohol drinking	N = 2179	N = 2168	N = 1769
HR per 100 mg/d (95% CI)	1.02 (0.74, 1.42)	0.85 (0.39–1.85)	1.04 (0.23–4.58)
*P* value	0.89	0.68	0.96
Current/ever alcohol drinking	N = 11034	N = 10997	N = 9776
HR per 100 mg/d (95% CI)	0.50 (0.28, 0.87)	0.50 (0.23–1.00)	0.46 (0.22–0.99)
*P*-value	0.016	0.05	0.05

^a^Cox’s proportional hazards model was performed with the SURVEYPHREG procedure to estimate

hazard ratios and 95% confidence intervals. Model 1: Adjusted for age; Model 2: Additionally adjusted for total energy intake; Model 3: Further adjusted for race, sex, BMI, waist to hip ratio, education attainment, household income, physical activity, smoking status, intake of calcium, or alcohol drinking.

^b^Unweighted N for each model differs due to some missing data for some covariates.

^c^*P* values for the interactions between magnesium intake (continuous) and hepatic steatosis (yes/no), drinking status (yes/no) for the specific mortality were 0.84 and 0.86 respectively.

## Discussion

Consistent with our hypothesis, we observed that high intake of magnesium was associated with a reduced risk of mortality due to liver disease in this NHANES III cohort, a nationally representative sample of the U.S. general population that included participants from all across the US. This observed inverse association was more apparent in those with fatty liver disease and among alcohol drinkers at baseline although the test for the interactions was not statistically significant. To our knowledge, this is the first study to investigate the association between magnesium intake and mortality due to liver disease.

Our finding that high magnesium intake was associated with a reduced risk of mortality due to liver disease is promising. The underlying mechanism is not entirely clear. However, the finding has very significant public health and clinical significace because it was reported in the National Health and Nutrition Examination Survey (NHANES), 1999–2000, that 79% of US adults do not meet the Recommended Dietary Allowance for magnesium^26^. One study observed that serum concentrations of magnesium were significantly reduced in patients with either alcoholic or non-alcoholic liver steatosis^14^. The findings are not surprising because of previous studies that found that people who regularly drink alcohol and those with type 2 diabetes are at high risk of magnesium deficiency^15–17^. For instance, 50% of people with type 2 diabetes had hypomagnesemia^19^. Studies conducted in the US and other Western populations show that low magnesium intake is associated with an elevated risk of insulin resistance^27,28^, systemic inflammation^29,30^, metabolic syndrome^31–33^, subclinical atherosclerosis and type 2 diabetes^34–37^. Since NAFLD is considered a characteristic of metabolic syndrome^6^, there is preliminary evidence indicating magnesium does have a role in liver disease despite an unclear mechanism.

Previous studies indicate that inflammation and insulin resistance play important roles in the progression of NAFLD^6–9^,alcoholic liver disease^10,11^ and hepatitis C and B^12,13^. Furthermore, randomized trials found magnesium treatment improved both inflammation and insulin resistance in participants at high risks of inflammation, insulin resistance, particularly those at high risk of magnesium deficiency. Therefore, supplements or high intakes of magnesium may prevent the progression of liver disease in those at high risk of magnesium deficiency, especially if they also have elevated inflammation levels and insulin resistance^22–25^. In this regard, it is plausible that adequate magnesium intake, which is related to improved insulin senstivity and inflammation, may slow development and progression of steatohepatitis and steatosis and in turn reduce the risk of mortality due to liver disease. This may provide a possible explanantion for the observation of an inverse association between magnesium and death from liver disease in the current study.

A strength of our study is that it is based on NHANES, a population-based study with a nationally representative sample. Although multiple 24-hour dietary recalls are used as a gold standard measure in nutritional epidemiologic studies, a one-time 24-hour dietary recall used in the current study may not have adequately captured long-term dietary intake of magnesium. Since inter-day variation in magnesium intake is random, any residual inter-day variation in the current study would lead to non-differential misclassification, which usually biases the result to the null. Thus, the true association between magnesium intake and mortality due to liver disease may be stronger than what we have observed. We cannot eliminate the possibility that the inverse associations with magnesium intake are due to redusidual confounding factors or healthy lifestyle in general. However, we have adjusted for physical activity and intake of calcium in all our analyses. Furthermore, in the same analysis, we did not find total intake of calcium to be related to the risk of death from liver disease. Thus, our finding for magnesium is unlikely to be due to confounding by healthy lifestyle in general because we have adjusted for physical activity and those who possess healthy behaviors are much more like to consume calcium supplementation with or without vitamin D than magnesium supplementation. We additionally adjusted for the intake of fiber in the model and found similar results (data not shown). We did not find any significant associations between the Ca:Mg intake ratio and risk of mortality due to liver disease. The association *per se* may not be accurate. For example, a high ratio could mean a high intake of calcium or low intake of magnesium. In our previous studies, we used the joint associations of Ca:Mg ratios with intakes of calcium or magnesium^38^. However, the power is not enough for this analysis in the current study. We also conducted an analysis and found that Mg/calories was not significantly related to the risk (data not shown). A nutrient density approach was proposed for energy-dense macronutrients, such as protein, carbohydrates and fats^39^. One possible explanation for the non-significant association could be that magnesium is not an energy dense macro-nutrient. Future studies are needed to confirm our findings. One weakness is that no additional assessments were conducted after the baseline evaluation of magnesium intake level. Also, since we used 24-hour dietary recall data instead of food frequency questionnaire data, we were not able to analyze the associations between specific foods rich in magnesium and calcium in relation to risk of mortality due to liver disease. In addition, the intake of magnesium and calcium from water has not been considered and this may lead to non-differential misclassification, which usually biases the results the null. Future studies are needed to investigate these associations.

In stratified analyses, we found the inverse associations seemed only of borderline significance among those with fatty liver or those who were alcohol drinkers at baseline. These findings are plausible because those with fatty liver at baseline are less likely to be alcohol drinkers as shown in Table 1. The interactions of magnesium intake with both fatty liver status and alcohol drinking status are not statistically significant. However, the non-significant interactions could, in part, be due to the small sample sizes in subgroup analyses. Future studies with larger sample sizes are needed to replicate the findings.

In conclusion, our findings suggest that high intake of magnesium may be associated with a reduced risk of mortality due to liver disease. However, further studies, including randomized trials, are warranted to confirm the findings.

## Methods

### Study Population and Outcomes

The NHANES III was conducted in the United States from 1988 through 1994 by the National Center for Health Statistics (NCHS) of the Centers for Disease Control and Prevention (CDC)^40^. The survey was approved by the institutional review board of the CDC, and all participants provided written informed consent to participate. Publically available data without personal identifiable information were used for the current study. All methods were performed in accordance with the relevant guidelines and regulations. It consisted of interview, medical examination, and laboratory data collected from a complex multistage, stratified, clustered probability sample representative of the civilian, noninstitutionalized population. In total, 33,994 persons aged 2 months and older participated in the survey. Participants were excluded if they were aged younger than 20 years old (15,169), with missing hepatic steatosis status data (4,969). We also excluded those with missing data on magnesium intake (352). Finally we included 13,504 participants in our study cohort. These participants were followed for mortality status from baseline until December 31, 2006. The median follow-up time was 14.6 years. Mortality outcomes were determined by using probabilistic linkage with the National Death Index (NDI). Cause of death coding for all U.S. deaths occurring prior to 1999 follows the 9^th^ revision of the International Statistical Classification of Diseases, Injuries and Causes of Death (ICD-9) guidelines, while all deaths after 1998 follows the 10^th^ revision of the International Statistical Classification of Diseases, Injuries and Causes of Death (ICD-10) guidenlines. Mortality due to liver disease included participants who died of malignant neoplasms of liver and intrahepatic bile ducts, chronic liver disease and cirrhosis.

### Nutrient Intake Assessments

Daily dietary intake data were obtained from one 24-hour dietary recall and 30-day supplement interviews, which were obtained at the baseline and were described in detail elsewhere^41^. Only dietary recall data with a status of ‘reliable’ were used in the analysis. Supplemental intake of magnesium was obtained from the response to a dietary supplement questionnaire. Total intake of magnesium was calculated by summing intakes from diet and supplements.

### Hepatic Steatosis

The ultrasound examination was recorded using a Toshiba Sonolayer SSA-90A and Toshiba video recorder among participants aged 20 to 74 years in NHANES III between 1988 and 1994. In 2009–2010, archived gall bladder ultrasound video images were reviewed to assess the presence of fat within the hepatic parenchyma using standard criteria. Using five criteria (liver to kidney contrast; brightness of the liver parenchyma; deep beam attenuation; echogenic walls in the small intrahepatic vessels; and the definition of the gallbladder walls), hepatic steatosis was categorized as normal, mild, moderate, or severe. In order to avoid potential overlap between mild and moderate hepatic steatosis, a categorization of hepatic steatosis as “yes” or “no” was generally used. “Yes” indicates moderate or severe hepatic steatosis, while “No” indicates the liver was normal or had mild hepatic steatosis^42^.

### Covariates

We considered a number of factors as potential confounding factors, including sex (male, female); race and ethnicity (non-Hispanic whites, non-Hispanic blacks, and other race); education (lower than high school education, high school diploma, and college graduate or above); ratio of poverty to income (PIR: ≤1, 1–3, >3); cigarette smoking status (never, former, current); alcohol drinking status (never, former, current); exercise (compared with most men or women of your age, you are less active, about the same, more active). Age, daily intakes of total energy (kcal), calcium (mg), magnesium (mg), body mass index (BMI) were included as continuous variables for adjustment in models.

### Statistical Analysis

We performed statistical analyses using the “Survey” procedure in SAS 9.4 software (SAS Institute, Cary, NC, USA) to estimate variance after incorporating the weights for the sample population in NHANES III cohort. For individuals who were not known to be deceased or who died from other causes, observations were censored at the date of death or December 31, 2011, whichever was earlier. Covariates were compared among the normal or mild hepatic steatosis group and the moderate to severe hepatic steatosis group to evaluate potential confounding factors using Rao-Scott chi-square test for categorical data and survey regression model for continuous variables. In addition to examining calcium as a potential confounding factor, we also examined whether intake of calcium was related to risk of mortality due to liver disease. Hazard ratios (HR) and (95% CIs) for the association between magnesium intake and mortality due to liver diease were estimated from Cox proportional hazard regression models fitted using PROC SURVEYPHREG procedure in SAS. HRs for mortality due to liver disease were estimated per every 100 mg increase in magnesium or calcium intake. Additionally we performed analyses stratified by hepatic steatosis and alcohol drinking status at baseline. P values are two-sided and were considered significant at P ≤ 0.05 in all analyses.
