# Author Correction: Magnesium intake and mortality due to liver diseases: Results from the Third National Health and Nutrition Examination Survey Cohort

**DOI:** 10.1038/s41598-019-42590-3

**Published:** 2019-05-01

**Authors:** Lijun Wu, Xiangzhu Zhu, Lei Fan, Edmond K. Kabagambe, Yiqing Song, Menghua Tao, Xiaosong Zhong, Lifang Hou, Martha J. Shrubsole, Jie Liu, Qi Dai

**Affiliations:** 1 Department of Digestive Diseases of Huashan Hosptial, Fudan University, Shanghai, 200040 China; 2 Division of Epidemiology, Department of Medicine, Vanderbilt University Medical Center, Nashville, TN 37203 USA; 3 Vanderbilt Ingram Cancer Center, Vanderbilt University School of Medicine, Nashville, TN 37232 USA; 4 Department of Oncology, Longhua Hospital, Shanghai University of Traditional Chinese Medicine, Shanghai, 200437 China; 5 Department of Epidemiology, Richard M. Fairbanks School of Public Health, Indiana University, Indianapolis, IN 46202 USA; 6 Department of Biostatistics and Epidemiology, University of North Texas Health Science Center, Fort Worth, TX 76107 United States; 7 Cancer Center, Beijing Shiji Tan Hospital, Capital Medical University, Bejing, 100038 China; 8 Department of Preventive Medicine, Feinberg School of Medicine, Northwestern University, 680 N. Lake Shore Drive, Suite 1400, Chicago, IL 60611 USA

Correction to: *Scientific Reports* 10.1038/s41598-017-18076-5, published online 20 December 2017

The original version of this Article contained an incorrect email address for Jie Liu, which was incorrectly listed as jieliu28@fudan.edu.cn. This error has now been corrected in the HTML and PDF versions of the Article.

